# A Single‐blind, Randomized, Single‐dose, Two‐sequence, Two‐period, Crossover Study to Assess the Bioequivalence between Two Oral Tablet Formulations of Rivaroxaban 20 mg in Healthy Mexican Volunteers

**DOI:** 10.1002/cpdd.1092

**Published:** 2022-05-05

**Authors:** Luis Genis‐Najera, Maria Elena Sañudo‐Maury, Trinifer Moquete

**Affiliations:** ^1^ Industrial Affairs Division Sanofi Mexico Ciudad de México Mexico; ^2^ Medical Division Sanofi Mexico Ciudad de México Mexico

**Keywords:** anticoagulants, bioequivalence, pharmacokinetics, rivaroxaban, safety

## Abstract

The objective of this study was to demonstrate the bioequivalence of 2 oral tablet formulations of rivaroxaban 20 mg in healthy Mexican volunteers under fed conditions. This phase I, single‐blind, single‐dose, randomized, two‐sequence, two‐period crossover study included 32 volunteers. Subjects were randomly assigned to one of two sequences: test formulation (single 20 mg dose) in the first period followed by the reference formulation (single 20 mg dose) in the second, or vice versa. Blood samples were collected predose and at predefined timepoints across a 48‐hour period after drug intake. Rivaroxaban plasma concentrations were measured using a validated high‐performance liquid chromatography‐tandem mass spectrometry method. Pharmacokinetic parameters included maximum plasma concentration (C_max_), area under the plasma concentration‐time curve from time zero to last measurable concentration and to infinity (AUC_0‐t_, AUC_0‐∞_), time to reach C_max_, and half‐life. Safety was evaluated through adverse‐event monitoring using subject interviews and recording of vital signs. The 90% confidence intervals for the test/reference geometric mean ratios of C_max_ (100.4%–112.7%), AUC_0‐t_ (96.5%–111.6%), and AUC_0‐∞_ (95.5%–109.5%) were within the bioequivalence acceptance range (80‐125%). Two adverse events (headaches) were recorded. Both formulations of rivaroxaban 20 mg tablets were bioequivalent and well tolerated in a healthy population of Mexican volunteers under fed conditions.

Anticoagulants have a central role in the prevention and treatment of thromboembolic disorders.^1^, ^2^ Interest in anticoagulation therapy has increased considerably over the last two decades, as reflected by a growing number of drugs in clinical development and a wide spectrum of licensed anticoagulants.^3^

Direct oral anticoagulants (DOACs) have been developed to address the shortcoming of established anticoagulant drugs, such as vitamin K antagonists.^4^ Rivaroxaban is an oral, selective, direct Factor Xa inhibitor.^5^

Rivaroxaban is rapidly absorbed in healthy fasting subjects, reaching maximum plasma concentrations (C_max_) approximately 2 hours after a single tablet intake (1.25‐80 mg).^6^ Under fed conditions, the median time to reach C_max_ (T_max_) ranges between 3 and 3.5 hours (10‐, 15‐, and 20‐mg tablets).^2^ Oral bioavailability for the 10‐mg tablet is high (80%–100%) regardless of fasting or fed conditions.^2^, ^7^ However, the bioavailability of the 20‐mg tablet is considerably reduced when administered without food (66%). Food restores the high oral bioavailability (≥80%) of rivaroxaban at this dose, increasing the area under the plasma concentration‐time curve (AUC) and C_max_ by 39% and 76%, respectively.^2^ As a result, the summary of product characteristics (SPC) of rivaroxaban recommends administration of the 20‐mg dose in a fed state. Given that the 15‐ and 20‐mg doses are used for the same indications, the SPC states that the former should also be taken with food to ensure consistent posology recommendations.^2^

Rivaroxaban exhibits a dual mode of elimination, with two‐thirds of the drug being subject to metabolic degradation in the liver and the remaining one‐third being eliminated as unchanged drug in the urine, mainly via active renal secretion.^4^ P‐glycoprotein and breast cancer resistance protein are the transporters involved in the active renal secretion of rivaroxaban.^4^, ^8^, ^9^ Rivaroxaban is metabolized via cytochrome P450 enzymes (CYP3A4/5 and CYP2J2) and CYP‐independent mechanisms.^4^ Unchanged rivaroxaban is the most important compound in plasma after administration. This drug has no major or active circulating metabolites.^4^ Under fed conditions, the half‐life (t_1/2_) of rivaroxaban 20 mg is 12.1 hours.^2^

DOACs have been commonly used in Mexico since 2008.^10^ Although the pharmacokinetic profile of rivaroxaban has been previously studied,^2^, ^11^, ^12^ no data are available in a Mexican population. This phase I trial was conducted to demonstrate the bioequivalence of 2 oral tablet formulations of rivaroxaban 20 mg in a selected population of healthy Mexican volunteers under fed conditions.

## Subjects and Methods

### Study Design

This was a phase I, single‐blind, single‐dose, randomized, two‐sequence, two‐period crossover study conducted in healthy volunteers at a single research center in Mexico, DEBBIOM, Desarrollos Biomédicos y Biotecnológicos de México, SA de CV. In line with rivaroxaban's prescribing information, which recommends administration of the 20‐mg dose with food,^2^ this study was conducted under fed conditions. Study periods were separated by a 7‐day washout interval to minimize potential carryover effects from the previous period. A standard 1:1 randomization approach was used to assign subjects to one of two sequences: test formulation (single 20‐mg dose) in the first period followed by the reference formulation (single 20‐mg dose) in the second, or vice‐versa.

The study adhered to the ethical principles of the Declaration of Helsinki, the International Conference on Harmonisation Good Clinical Practice Guidelines, and the Mexican NOM 177‐SSA1‐2013, which establishes the requirements for bioequivalence studies. The study protocol (F1/CCGE‐P01) was approved by the Ethics Committee of the study center (Comité de Ética en Investigación de Desarrollos Biomédicos y Biotecnológicos de México S.A. de CV) on April 17, 2018, and the Comisión Federal para la Protección contra Riesgos Sanitarios (COFEPRIS) on May 28, 2018.

### Subjects

Male and female volunteers aged 18 to 55 years, with a body mass index (BMI) between 20 and 27 kg/m^2^, and in good health based on medical history, physical examination, and laboratory screening were eligible to participate. Main exclusion criteria included severe hepatic impairment, hematopoietic disease, history of gastrointestinal bleeding or renal disease, known hypersensitivity to the active principle or any excipient or rivaroxaban, pregnancy, positive urine test for drugs of abuse, history of drug use and/or alcoholism, and use of tobacco, alcohol, or any medication within the 24 hours prior to study start. All volunteers provided written informed consent before participation.

### Study Drugs

The reference and test formulations used in this study were Xarelto 20‐mg tablets (lot no. BXHZ5T1, expiration date October 2019; Bayer de México, SA de CV, Mexico) and Rivaroxaban 20‐mg tablets (lot no. 8MXA002; expiration date July 2020; Sanofi Aventis de Mexico, S.A. de CV, Mexico), respectively.

### Study Conduct and Procedures

In both study periods, subjects were admitted to the study center on the day prior to study drug administration and underwent further screening and physical examination to confirm eligibility. After an overnight stay, eligible subjects were given a standardized breakfast 0.5 hours before drug intake, to be consumed within 25 minutes. The breakfast consisted of a cup of rice cereal, one banana, one apple, and 250 mL of milk, equivalent to approximately 380 calories. Study drugs (single 20‐mg tablet of test or reference formulation) were administered along with 250 mL of noncarbonated water at around 8 am. The subjects were under continuous medical supervision while at the study center.

At each study period, 16 blood samples were collected into heparinized vacuum tubes at the following time points: before drug intake (0 hours) and at 0.25, 0.5, 0.75, 1, 1.5, 2, 2.5, 3, 3.5, 4, 6, 8, 12, 24, and 48 hours postadministration. Vital signs were measured at admission to the study center, at the time of the first blood sample collection, and at 1, 1.5, 2, 4, 8, and 11.75 hours after dosing.

### Analytic Methodology

Blood samples were centrifugated at 3500 rpm for 10 minutes at room temperature to obtain plasma samples, which were stored in a deep freezer at ‐70 ± 20°C until analysis.

Rivaroxaban plasma concentrations were measured using a high‐performance liquid chromatography‐tandem mass spectrometry (HPLC‐MS/MS) method. The bioanalytical method was validated according to the acceptance criteria defined in NOM‐177‐SSA1‐2013 for system suitability, selectivity, linearity, precision, and accuracy within‐ and between‐run, matrix effect, absolute recovery, sample stability, and carryover effect.•

Rivaroxaban was extracted from human plasma through the liquid–liquid extraction method using methyl tert‐butyl ether as extraction solvent and letrozole as internal standard. The chromatographic separation was performed on a Zorbax Eclipse Plus C18 50 × 4.6 mm column. The mobile phase consisted of methanol/formic acid 0.088% (80:20). The flow rate was 0.6 mL/min. The autosampler and column temperatures were maintained at 5 and 40°C, respectively. The compounds were detected in positive electrospray ionization with the multiple reaction monitor (MRM) mode. The MRM transitions were *m*/*z* 436.3–145.1 for rivaroxaban and *m*/*z* 286.2–271 for the internal standard. The linear range was 2.0–400.0 ng/mL for rivaroxaban. Analytical data were processed using the Analyst 1.6 software. The within‐run and between‐run coefficients of variation (CVs) for precision and accuracy were lower than 10% for nominal concentrations and lower than 16% for the lower limit of quantification.

### Safety Assessments

The safety of the test and reference formulations was evaluated through adverse‐event (AE) monitoring. AEs noted by the investigator or reported by the subjects, spontaneously or during direct interviews, were recorded. Additionally, vital signs (blood pressure, heart rate, respiratory rate, and body temperature) were assessed at various timepoints throughout the study.

### Statistical and Pharmacokinetic Analyses

The sample size was estimated based on the results from a previous bioequivalence study of rivaroxaban.^13^ The intrasubject CVs were determined for the ln‐transformed pharmacokinetic parameters reported by Rouini et al (C_max_, AUC_0‐t_, and AUC_0‐∞_),^13^ with the highest one (26.1%, for C_max_) being used for the sample size calculation. Based on the Shein–Chung Chow equation for crossover design,^14^ a total of 30 subjects were required to demonstrate the bioequivalence of the test and reference formulations with a power of at least 90%. Two additional subjects were added to account for possible drop‐outs, resulting in a total sample size of 32 healthy volunteers.

All randomized subjects who received both rivaroxaban formulations during the study were included in the pharmacokinetic and statistical analyses. Safety was evaluated based on data from all randomized subjects who received at least one dose of rivaroxaban.

Pharmacokinetic parameters were derived from plasma concentration–time data using a noncompartmental approach with a log‐linear terminal phase assumption and summarized by descriptive statistics, namely arithmetic and geometric means, standard deviation, median, minimum, maximum, and CV. These parameters included C_max_, AUC from time zero to last measurable concentration (AUC_0‐t_), AUC from time zero to infinity (AUC_0‐∞_), T_max_, and t_1/2_.

An analysis of variance including sequence, subjects within sequence, period, and formulation was carried out for ln‐transformed C_max_ and AUC_0‐t_. The 90% confidence intervals (CIs) of the test/reference geometric mean ratios (GMRs) for these parameters were then determined based on logarithmically transformed data. Bioequivalence between the test and reference formulations of rivaroxaban was demonstrated if the 90%CIs for ln C_max_ and AUC_0‐t_ were within the acceptable range of 80%–125%. Although not required to demonstrate bioequivalence, 90%CIs were also computed for AUC_0‐∞_.

Pharmacokinetic analysis was performed with Phoenix 64 WinNonlin 7.0.

## Results

### Subject Disposition and Baseline Characteristics

A total of 32 healthy volunteers (29 females and 3 males) were enrolled and randomized, all of whom received at least one dose of rivaroxaban. Thirty‐one subjects completed the study; one subject withdrew for personal motives and did not participate in the second period. Therefore, all 32 subjects were valid for the safety evaluation, while 31 subjects were included in the pharmacokinetic analysis.

Subjects’ baseline characteristics are described in Table 1. Mean age was 30.0 (±9.6) years, and mean BMI was 24.0 (±1.7) kg/m^2^.

**Table 1:** Healthy Volunteer Baseline Characteristics

**Baseline characteristic**	**Total (*n* = 32)**
Sex (female)	29 (90.6%)
Age (years)	30.0 ± 9.6
Weight (kg)	61.7 ± 7.0
Height (m)	1.60 ± 0.06
Body mass index (kg/m^2^)	24.0 ± 1.7
Systolic blood pressure (mmHg)	101.5 ± 9.4
Diastolic blood pressure (mmHg)	66.4 ± 7.3
Heart rate (times per minute)	76.3 ± 10.2
Respiratory rate (times per minute)	16.4 ± 1.2
Temperature (°C)	36.5 ± 0.3

Results are displayed as *n* (%) or mean ± standard deviation.

### Pharmacokinetics Parameters

**Figure 1:**
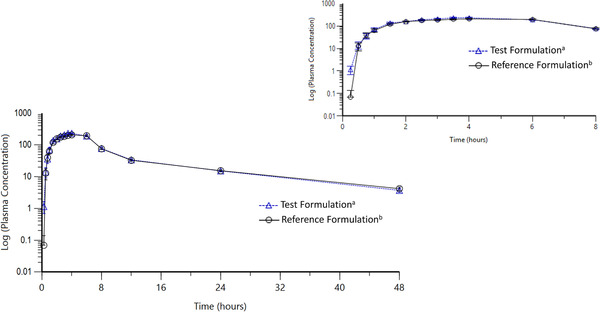
Mean (± standard error) plasma concentration‐time profiles of rivaroxaban following a single 20‐mg oral dose of the test and reference formulations. Upper right inset shows 0–8 hours on an expanded time scale. Triangles indicate rivaroxaban 20 mg (Sanofi Aventis de Mexico S.A. de C.V.), and circles indicate Xarelto 20 mg (Bayer de México, SA de C.V.).

Pharmacokinetic parameters after a single‐dose administration under fed conditions were similar for the test and reference formulations (Table 2). Mean C_max_, AUC_0‐t_, and AUC_0‐∞_ were, respectively, 261 ng/mL, 1948 ng h/mL, and 2045 ng h/mL for the test formulation and 246 ng/mL, 1885 ng h/mL, and 2001 ng h/mL for the reference formulation. Median T_max_ was 3.5 hours for the test and 4.0 hours for the reference formulations. Mean rivaroxaban plasma concentration versus time curves for each formulation are presented in Figure 1.

**Table 2:** Pharmacokinetic parameters after a single 20‐mg oral dose of the test and reference formulations

**Pharmacokinetic parameter**	**Test formulation** **^a^**	**Reference formulation** **^b^**
C_max_ (ng/mL)		
Arithmetic mean	261	246
Geometric mean	255	240
SD	56	58
CV, %	21	24
AUC_0‐t_ (ng • h/mL)		
Arithmetic mean	1948	1885
Geometric mean	1891	1827
SD	460	471
CV, %	24	25
AUC_0‐∞_ (ng h/mL)		
Arithmetic mean	2045	2001
Geometric mean	1990	1950
SD	464	459
CV, %	23	23
t_1/2_ (hours)		
Arithmetic mean	10.58	11.96
Geometric mean	9.64	10.83
SD	4.81	5.49
CV, %	45	46
T_max_ (hours)		
Median	3.50	4.00
Range	1.50–6.00	0.75–6.00

AUC_0‐t_, area under the plasma concentration‐time curve from zero to last measurable concentration; AUC_0‐∞_, area under the plasma concentration‐time curve from zero to infinity; C_max_, maximum plasma concentration; CV, coefficient of variation; SD, standard deviation; T_max_, time to reach maximum plasma concentration; t_1/2_, half‐life.

^a^ Rivaroxaban 20 mg (Sanofi Aventis de Mexico S.A. de C.V.).

^b^ Xarelto 20 mg (Bayer de México, SA de C.V.).

### Bioequivalence

Analysis of variance for ln‐transformed PK parameters (C_max_, AUC_0‐t_, and AUC_0‐∞_) showed no statistically significant effect of variation due to sequence or formulation (*P* ≥ .05). The effect of period was significant for C_max_ (*P* = .0087), but not for AUC_0‐t_ or AUC_0‐∞_. The test/reference GMRs (90%CIs) for C_max_, AUC_0‐t_, and AUC_0‐∞_ were 106.4% (100.4%–112.7%), 103.8% (96.5%–111.6%), and 102.3% (95.5%–109.5%), respectively. All the 90%CIs for these PK parameters were within the bioequivalence range (80%–125%) (Table 3).

**Table 3:** The 90%CIs of ln‐transformed parameters of the test and reference formulations

Parameter	GMR test^a^/reference^b^ (%)	90%CI		Power
		Lower	Upper	
ln C_max_	106.4	100.4	112.7	1.00
ln AUC_0‐t_	103.8	96.5	111.6	0.99
ln AUC_0‐∞_	102.3	95.5	109.5	0.99

AUC_0‐t_, area under the plasma concentration‐time curve from zero to last measurable concentration; AUC_0‐∞_, area under the plasma concentration‐time curve from zero to infinity; CI, confidence interval; C_max_, maximum plasma concentration; ln, logarithm; GMR, geometric mean ratio. For this publication, the calculation of the GMR and 90%CI for AUC_0‐t_ and AUC_0‐∞_ parameters were calculated using LOQ = 0, but the original data submitted to the Mexican Health Authorities considered the values below of LOQ = “missing” for the calculation of GMR and 90%CI for AUC_0‐t_ and AUC_0‐∞_ parameters. This statistical consideration does not have any impact on the results since the difference is minimal, after the point. The results were AUC_0‐t_, GMR 103.8439 with 90%CI 96.6130 and 111.6161; AUC_0‐inf_, GMR 102.3352 with 90%CI 95.5805–109.5673.

^a^ Rivaroxaban 20 mg (Sanofi Aventis de Mexico S.A. de C.V.).

^b^ Xarelto 20 mg (Bayer de México, SA de C.V.).

### Safety

No serious, severe, unexpected AEs or AEs leading to study discontinuation were observed in this trial. Two AEs (headache) (6.3%) were recorded among the 32 enrolled healthy volunteers, one after administration of the test formulation and the other after administration of the reference formulation. Both AEs met adverse drug reaction criteria. The AEs related to the test and reference formulations were of moderate and mild intensity, respectively. Both AEs were transient and resolved without sequelae.

## Discussion

The present study was designed to assess the bioequivalence of a single 20‐mg dose of 2 oral tablet formulations of rivaroxaban in healthy Mexican volunteers under fed conditions. The bioequivalence of the test and reference formulations with respect to both the rate and extent of absorption was confirmed. This is evidenced by the results showing that the 90%CIs for the ln‐transformed ratios of C_max_ and AUC_0‐t_ fell within the bioequivalence acceptance range (80%–125%). Although not required to demonstrate bioequivalence, the same conclusion can be drawn for AUC_0‐∞_. As rivaroxaban exhibits a dose‐proportional pharmacokinetic profile for doses of 10, 15, and 20 mg under fed conditions,^2^ our findings are likely applicable to lower rivaroxaban doses.

Previous studies have also evaluated the pharmacokinetic parameters of rivaroxaban 20‐mg tablets after a single‐dose administration in healthy subjects under fed conditions.^2^, ^15^, ^16^, ^17^ In these studies, mean C_max_ and AUC ranged from 272 to 351 ng/mL and from 2021 to 2644 ng h/mL, respectively. Our results, for both test and reference formulations, fall near the lower end of these ranges. Data on T_max_ align with those from prior studies, which found median values between 3.0 and 4.0 hours.

Our sample was predominantly comprised of female volunteers (90.6%). Sex has been shown to have no significant influence on the pharmacokinetics of rivaroxaban in healthy volunteers under fed conditions.^18^ Thus, similar results on pharmacokinetic parameters would likely have been found if the proportion of male and female volunteers had been more balanced.

Regardless of the formulation administered, our data show that rivaroxaban 20 mg is well tolerated among healthy subjects. This is reflected by the fact that only two subjects (6.3%) developed AEs and that no serious, severe AEs or AEs leading to study discontinuation were recorded during the study. Our findings are in line with previous studies that reported low rates of treatment‐emergent AEs following a single 20 mg dose of rivaroxaban in healthy subjects, all of which were of mild or moderate severity.^2^ Headache, the only type of AE recorded in this study, is listed on the SPC of Xarelto as a common adverse reaction and has been reported in the literature after administration of this dosage strength.^2^

## Conclusion

This single‐dose study demonstrated the bioequivalence of the test and reference formulations of rivaroxaban 20‐mg tablets in a healthy population of Mexican volunteers under fed conditions. Both formulations were well tolerated.

## Funding

This study was funded by Sanofi Mexico.

## Conflicts of Interest

All authors are Sanofi Mexico employees and may hold shares and/or stock options in the company. The authors have no other potential conflicts of interest relevant to this study.

## Author Contributions

L.G.N., M.E.S.M., and T.M. performed the data analysis and interpretation and statistical analysis and wrote, revised, and approved the manuscript.
